# The Impact of a Home Visiting Program on First‐Time Parents’ Parental Self‐Efficacy in Norway and Associations between Parental Self‐Efficacy, Depressive Symptoms and Perception of Sleep

**DOI:** 10.1111/phn.70098

**Published:** 2026-02-28

**Authors:** Kristin Marie Sæther, Milada Cvancarova Hagen, Bettina Holmberg Fagerlund, Kari Glavin, Malene Brekke, Beate Solberg, Anne‐Martha Utne Øygarden, Nina Jøranson

**Affiliations:** ^1^ Faculty of Health Sciences VID Specialized University Oslo Norway; ^2^ Oslo Metropolitan University Oslo Norway; ^3^ Faculty of Social and Health Sciences University of Inland Elverum Norway

**Keywords:** child health service, first‐time parents, home visit, parental self‐efficacy, universal family intervention

## Abstract

**Objective:**

To examine the impact of the New Families (NF) home visiting program on first‐time parents’ parental self‐efficacy (PSE) assessed at six weeks and three months postnatally and to explore associations between PSE and the selected possible predictors “depressive symptoms” and “perception of sleep”.

**Design:**

A prospective nonrandomized controlled trial.

**Sample:**

The intervention group (*N* = 142 mothers/121 fathers at baseline) received NF and standard care (traditional child health program). The control group (*N* = 86 mothers/76 fathers) received standard care.

**Measures:**

PSE was measured by the Perceived maternal parental self‐efficacy tool (PMP S‐E).

**Results:**

In a socioeconomic low‐risk sample, we found a statistically significant positive impact of NF on fathers' PSE at six weeks. No statistically significant impact was detected among fathers at three months or at any time point among mothers. Depressive symptoms and perception of insufficient sleep were associated with lower PSE in mothers.

**Conclusion:**

NF shows potential for reaching out to fathers and should be further investigated because there is a need for interventions supporting fathers postnatally. Despite the lack of impact on mothers’ PSE, further investigation of how to reach mothers with depressive symptoms and sleep issues would be of interest.

**Clinical trial registration**: ClinicalTrials.gov (NCT04162626).

## 1 Background

Becoming a parent is described as a complex and multifaceted transition (Sæther et al. 2023). The World Health Organization (WHO 2022) highlights that a positive postnatal experience provides a “platform for improved short‐ and long‐term health and well‐being”. Elements that support this experience are receiving consistent information, reassurance, and support from motivated health workers in a resourced and flexible health system which recognizes, and respects needs and cultural context (WHO 2022).

In Norway, families with children between 0 and 5 years are offered support through a national Child Health Service (CHS). The service is used by about 99% of the eligible population (Statistics Norway 2023), is free of charge, regulated by law, and guided by national best practice recommendations (Norwegian Directorate of Health 2017). A universal child health program with 14 time‐specific consultations is offered to all families by public health nurses (PHNs). The first consultation in the program is a home visit 7–10 days after discharge from hospital following childbirth. Subsequent consultations are located in local child health clinics (CHCs). Additionally, the child receives a health check by a doctor at 6 weeks, 6 months, 1 year and 2 years after birth (Norwegian Directorate of Health 2017).

To strengthen the CHS as a universal, early, and tailored service, the supplementary New Families (NF) home visiting program was developed by the City of Oslo between 2013 and 2016 and stepwise implemented in all city districts of Oslo (City of Oslo 2018; Leirbakk et al. 2018, 2019, 2023). NF has a salutogenic perspective based on Antonovsky's concepts of sense of coherence and general resistant resources and Bandura's self‐efficacy (SE) theory (Antonovsky 1987; Bandura 1997, City of Oslo 2018). Additionally, it is recommended to use motivational and emphatic communication techniques to create a good relational environment (Brudal 2014; City of Oslo 2018; Miller and Rollnick 2023).

The NF program consists of repeated home visits conducted by PHNs from the 28^th^ week of pregnancy until the child is 2 years old. However, the number and agenda of the visits are not standardized, but rather tailored to the parents’ needs (City of Oslo 2018). The program is voluntary, free of charge and a universal offering for all first‐time parents and parents expecting their first child together and/or their first child in Norway irrespective of risk factors. NF exemplifies *proportionate universalism* whereby the key principle is *provision for all—distributed according to need*. This is a public health strategy combining a degree of *positive selectivism* within a universal framework and is an alternative to high risks strategies (Carey, et al. 2015; Cowley et al. 2015; Marmot et al. 2010).

Enhancing parental self‐efficacy (PSE) is a common aim in health‐promoting interventions, and PSE is frequently used as an outcome measure in evaluation studies (Brekke et al. 2023; Liyana Amin et al. 2018; Shorey et al. 2015; Wittkowski et al. 2017). PSE is defined as parents’ “beliefs or confidence in their ability to successfully carry out parenting tasks” and is captured as a domain‐specific concept under Bandura's SE theory (Vance and Brandon 2017, p. e30).

In a systematic review of universal parent education interventions provided to first‐time parents (Liyana Amin et al. 2018), duration and consistency of support were the most crucial factors in enhancing PSE. Further highlighted were universal interventions’ opportunities to reach out to first‐time parents at risk of difficulties adjusting to parenthood and to target fathers.

In Norway, fathers are expected to take a minimum of 15 weeks of parental leave (The Norwegian Digitalisation Agency 2025). However, recent qualitative studies highlight that fathers neither feel included nor satisfied with the support from the regular CHS (Høgmo et al. 2021; Solberg and Glavin 2018). This indicates an incongruity between societal expectations of the fathers’ participation in childcare and their perceived support. Furthermore, functional coparenting, which refers to parents collaborating well in their parental roles, is crucial in enhancing PSE by providing their partner with support instead of undermining it (Feinberg 2002, 2003). Moreover, interventions that facilitate coparenting are found to particularly impact fathers’ PSE (Favez et al. 2016; Feinberg et al. 2020). In light of this and a general lack of research on fathers (Davison et al. 2017), we address both mothers and fathers in this study.

The main aim of this study was to examine the impact of NF on first‐time mothers’ and fathers’ PSE, assessed at six weeks and three months postnatally by comparing an intervention group receiving both NF and standard care (the traditional child health program) with a control group receiving standard care only. Further, if no impact of NF was found at six weeks (separately, in the sample of mothers and fathers), we aimed to explore possible associations between PSE at six weeks and the selected possible predictors “depressive symptoms” and “perception of sleep.”

## 2 Methods

The current study is a prospective nonrandomized controlled trial with a parallel group design, using data from the overall research project “NF—Innovation and Development of the CHS in Oslo” (ClinicalTrials.gov identifier: NCT04162626). The overall research project seeks to evaluate the NF home visiting program's impact on different outcomes like quality of life in mothers (Brekke et al. 2023) and depressive symptoms in fathers (Solberg et al. 2024). The current study focuses on the outcome of PSE in both mothers and fathers. We report in accordance with the Transparent Reporting of Evaluations with Nonrandomized Designs (TREND) Statement checklist (Des Jarlais et al. 2004).

### 2.1 Study Setting and Sampling Procedure

The City of Oslo comprises 15 city districts. Randomization of districts was not possible because the intervention had already been rolled out in some districts when the overall research project started. A department in the City of Oslo selected and matched five city districts according to their population composition, immigrant proportion, birth statistics, and work participation. Three districts served as the intervention group, and two districts served as the control group. The questionnaires were translated from Norwegian into nine languages to aid recruitment of parents of diverse ethnical backgrounds. A midwife or a clinic secretary recruited participants attending pregnancy check‐ups at the CHCs in the selected districts between October 2018 and December 2019.

### 2.2 Inclusion and Exclusion Criteria

Participants were eligible if they lived in one of the chosen districts, were to become a mother for the first time or were to be the father (not necessarily first‐time father) of the expected child and could communicate in Norwegian or any of the nine languages: English, Arabic, Lithuanian, Pashto, Polish, Somali, Tamil, Turkish or Urdu. The exclusion criterion was multiparous women.

### 2.3 Control Conditions and Study Intervention

**FIGURE 1:**
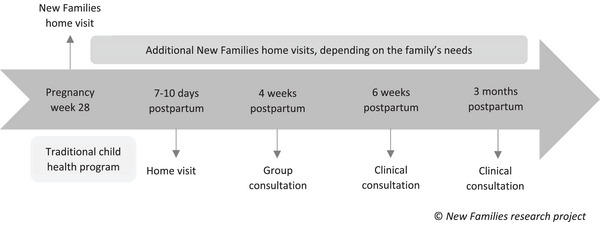
Timeline of the NF home visiting program in the context of the traditional child health program.

The control group received standard care through the traditional child health program, see Figure 1. During the first three months, which is the study period of this study, standard care offered a home visit by a PHN around 7–10 days postnatally followed up by three consultations with the same PHN at the CHC, including a group consultation. At six weeks standard care also included a health check by a doctor at the CHC. According to the best practice recommendations (Norwegian Directorate of Health 2017), vaccination, health checks, health information, and parental guidance are recommended for each specific consultation.

The intervention group received both NF and standard care. In the intervention districts, NF was integrated into the traditional child health program and delivered by the same PHN to achieve relational continuity. Expectant parents in the intervention group were offered a home visit around the 28^th^ week of pregnancy by the PHN, who subsequently followed up postnatally. The home visit was scheduled for one to one and a half hours. The PHNs conducted the home visits based on a manual, mandatory theoretical training, and guidance from a trained PHN (City of Oslo 2018). Besides starting a relationship between the parents and their PHN during the prenatal visit, information about the CHS's traditional child health program postnatally was provided. In addition, the PHNs were encouraged to address themes like *expectations and dreams of becoming a parent*, *reflections of the parents’ own childhood*, and *the impending birth* (City of Oslo 2018). Further, the number, frequency, and agenda of home visits from the NF program depended on the individual family's needs. The CHCs received additional funding to employ enough PHNs to deliver NF. Figure 1 illustrates the service delivered through NF and standard care.

### 2.4 Data Collection

The data collection for the current study lasted from October 2018 to June 2020. The recruited participants were contacted via SMS or telephone by a researcher for confirmation of participation. An information letter, a consent template, and the first questionnaire package were then sent by mail. For this study, data were collected at three time points: the 28^th^ week of pregnancy (T1), six weeks postnatally (T2), and three months postnatally (T3). However, the primary outcome (PSE) was only collected at T2 and T3. We were not allowed to collect data on nonresponders at T1 due to general data protection regulation laws.

Demographic data like age, income, marital status, education level, and nationality were collected at T1. In addition, previous and present mental health conditions, complications during pregnancy (mothers), relationship satisfaction, perception of sleep, depressive symptoms, and being a first‐time or multiparous father were assessed. Perception of sleep and depressive symptoms were also assessed at T2.

### 2.5 Instruments

The primary outcome, PSE, was measured by the “Perceived maternal PSE tool” (PMP S‐E) (Barnes and Adamson‐Macedo 2007). This instrument is domain‐specific and consists of 20 questions divided into four factors: caretaking procedures, evoking behaviour(s), reading behaviour(s) or signalling, and situational belief. All questions have four response options from 1 to 4 (strongly disagree, disagree, agree, strongly agree) and PSE is measured by summing up the 20 questions. This makes a possible score between 20 and 80. A higher score indicates a higher level of PSE. Barnes and Adamson‐Macedo (2007) reported a Cronbach's alpha coefficient of  0.91. In the current study, Cronbach's alpha coefficients were respectively  0.94/0.94 at T2/T3 for mothers and  0.91/0.93 for fathers. The PMP S‐E tool was translated and re‐translated to Norwegian by professional translators.

The PMP S‐E tool was originally intended for hospitalised mothers with relatively healthy preterm babies (Barnes and Adamson‐Macedo 2007) but has been validated to be used among mothers with full‐born, healthy babies between 0 and 12 months postnatally (Kahya and Uluc 2021; Monteiro et al. 2022; Vargas‐Porras et al. 2020), and is used in studies targeting both parents (Fong et al. 2018). The instrument is one of 34 instruments measuring PSE in parents with children 0–18 years and is rated second highest in psychometric and administrative properties (Wittkowski et al. 2017).

Depressive symptoms were measured by the Edinburgh Postnatal Depression Scale (EPDS), originally developed by Cox et al. (1987). The scale consists of 10 questions with a four‐point Likert scale (0–3), making a sum score from 0 to 30, and a higher score indicates a higher level of depressive symptoms. Eberhard‐Gran et al. (2001) validated the scale for postnatal women in Norway and recommended a cut‐off at 10, where EPDS ≥ 10 indicates depressive symptoms. In the present study, Cronbach's alpha coefficients of EPDS at T1 and T2 were for mothers  0.83/0.81 and fathers  0.74/0.80, considered sufficient internal reliability. Despite the lack of research on fathers, EPDS is found to be the most extensively assessed instrument measuring fathers’ depressive symptoms (Berg et al. 2022).

The Relationship Assessment Scale (RAS) was used to measure relationship satisfaction between mothers and fathers. This measurement consists of seven questions with a five‐point Likert scale (1–5), and the total score ranges from 7 to 35, where higher scores indicate greater relationship satisfaction (Hendrick 1988). The RAS measurement was translated and re‐translated to Norwegian by professional translators. Vaughn and Matyastik Baier (1999) reported a Cronbach's alpha coefficient of  0.86, and in the current study, Cronbach's alpha coefficient at T1 was for mothers  0.86 and for fathers  0.81, considered sufficient internal reliability.

### 2.6 Data Analyses

The overall research project was powered to assess possible differences between the intervention and the control group regarding depressive symptoms measured with EPDS (Cox et al. 1987). In collaboration with a statistician, the required sample size in the overall project was calculated to 64 participants in each group. This calculation was based on a statistical power of  0.80, an alpha level of  0.05, and an anticipated effect size of  0.50. Power calculation was not performed based on the outcome of this study, hence the number of respondents eligible and willing to participate during the study period determined the sample size.

Descriptive statistics were used to describe the sample characteristics. Categorical data were described as counts and percentages, and continuous variables with median and minimum and maximum values. Crude comparisons between pairs of variables were performed using the Mann‐Whitney‐*U* test (continuous variables) and the chi‐square (*χ* ^2^) test (categorical data). Regarding the chi‐square (*χ* ^2^) test for 2 x 2 tables, we report continuity corrected values, and when there are small numbers (5 or less) in some cells, we report Fisher's Exact Test 2‐sided *p*‐values. Variables that reached *p*‐values < 0.05 in univariate analyses were included in the multivariate model. In addition, we chose to include “Perception of sleep” in fathers in further analyses despite a continuity corrected *p*‐value of 0.052 because both Pearson chi‐square (*χ* ^2^) *p*‐value and Fisher's Exact Test 2‐sided *p*‐value were statistically significant. Further, we tested for possible baseline differences between responders and nonresponders at T2 and T3 on the variables of age, education, and family income.

The levels of PSE at T2 and T3 were described with mean and median, and to detect possible differences between the intervention and the control group, we fitted multivariate linear regression models. In the first model, the dependent variable was PSE at T2, and in the second model, the dependent was PSE at T3. Both models were adjusted for baseline differences between the intervention and the control group to eliminate possible confounding. The results are presented as regression coefficients B with 95 % confidence intervals (CI). The CIs were constructed using bootstrapping with 1000 repetitions. In addition, effect sizes (ES) were calculated and interpreted as small = 0.20, medium = 0.50, and large = 0.80 (Cohen's *d*).

Participants with one or several missing items of the PMP S‐E tool were excluded from the main analyses. The percentages of responders with no missing values at T2 and T3 were for mothers 89.7% (*n* = 165) and 97.0% (*n* = 162), and for fathers, 90.8% (*n* = 139) and 90.4% (*n* = 122). To assess if missingness would influence our results, we conducted sensitivity analyses and replicated the main analyses by imputing the missing values with the highest (4) and the lowest (1) value. In addition, we conducted a sensitivity analysis to assess the possible impact of the Covid‐19 pandemic.

As no statistically significant differences in PSE between mothers in the intervention group and the control group were found, we wanted to explore possible associations between PSE at T2 in the whole sample of mothers and the selected variables: “perception of sleep” at T1 (enough/not enough), “depressive symptoms” (EPDS ≥ 10) at T2 (yes/no), and “perception of sleep” at T2 (enough/not enough). “Perception of sleep” at T1 was selected as this variable reached the level of statistical significance and was thus associated with the level of PSE at T2 in the main analysis of mothers. “Depressive symptoms” and “perception of sleep” at T2 were selected because previous quantitative studies showed significant associations between PSE and these variables (Albanese et al. 2019; Carroll et al. 2024; Leahy‐Warren et al. 2012), as well as the variables were described as related to PSE in qualitative studies (Sæther et al. 2023). We performed a multivariate linear regression with the backward selection method. The variable “perception of sleep” at T1 (enough/not enough) was not statistically significant and we removed it from the model. We then performed a multivariate linear regression with the variables “depressive symptoms” (EPDS ≥ 10) at T2 (yes/no) and “perception of sleep” at T2 (enough/not enough), and constructed the CIs using bootstrapping with 1000 repetitions and calculated ES.

All statistical analyses were performed according to the intention to treat principle (Polit and Beck 2021), indicating that all participants in both groups were included irrespective of their use of standard care and/or the NF intervention. The analyses were performed separately for mothers and fathers and were checked by a statistician in our research group. All analyses were considered exploratory, so no correction for multiple testing was done. All tests were two‐sided, and *p*‐values < 0.05 were considered statistically significant. All analyses were conducted using SPSS, version 28, on a secure platform for Sensitive Data (University of Oslo 2023).

### 2.7 Ethical Considerations

Written informed consent to participate in the study was collected from all participants. Participation was voluntary, and participants could withdraw without giving a reason. All data were treated as confidential, and participant anonymity was guaranteed. The overall research project was approved by the Regional Committees for Medical and Health Research Ethics (reference no: 2018/1378) and the Norwegian Agency for Shared Services in Education and Research (project number: 735207) and was registered in ClinicalTrials.gov (NCT04162626). The study was designed and conducted in accordance with the Helsinki Declaration (World Medical Association 2013) and Artificial Intelligence (AI) has not been used.

## 3 Results

**FIGURE 2:**
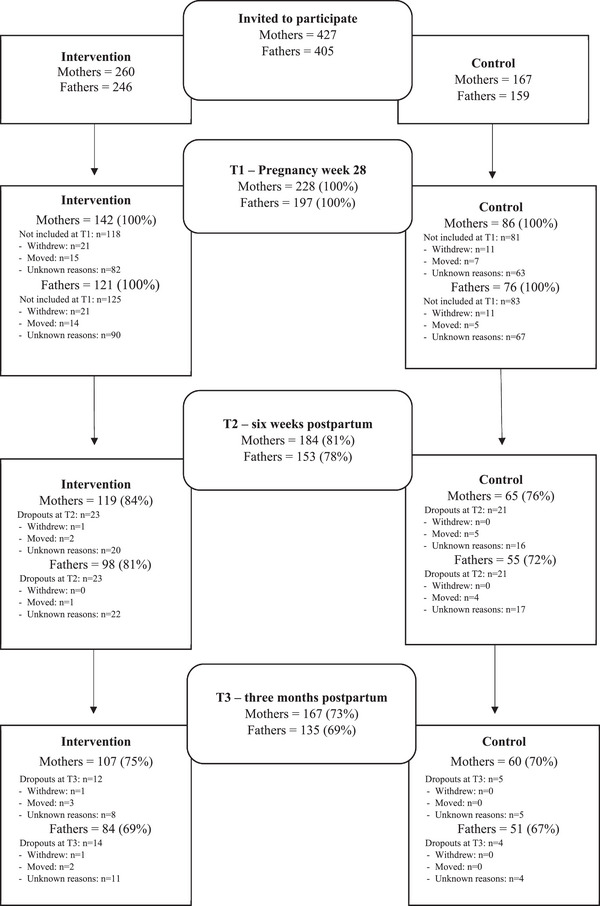
Flowchart.

In total, 427 first‐time mothers and 405 fathers were invited to participate in the study, and 228 (53.4%) mothers and 197 (48.6%) fathers accepted the invitation and were enrolled, resulting in a total response rate of 51.1%. The response rates of mothers and fathers at each time point are presented in the flowchart separately for the intervention and control groups, see Figure 2. When comparing responders and nonresponders (lost to follow up from T1) at T2 and T3 for the variables age, income, and education, we found no statistically significant differences in the sample of fathers nor in the intervention group of mothers. However, in the control group of mothers, the responders at T2 had statistically significantly higher education, and at T3, they were statistically significantly older than the nonresponders.

### 3.1 Characteristics of the Sample

The participants completed the questionnaires in Norwegian, English, or Arabic, where 95.6% (*n* = 218) of the mothers and 94.9% (*n* = 187) of the fathers answered in Norwegian at T1. All mothers and 91.9% (*n* = 181) of the fathers were first‐time parents. Most parents were between 29 and 35 years old, most were cohabiting or married, had a high education and income level, and were native Norwegians. Depressive symptoms (EPDS ≥ 10) occurred in 17.5% (*n* = 40) of mothers and 3.1% (*n* = 6) of fathers. The intervention group and the control group showed statistically significant differences in the variables of family income and perception of sleep at T1. Mothers and fathers in the intervention group reported higher income and lower perception of enough sleep compared to the control group (Table 1).

**TABLE 1:** Characteristics of the sample at baseline.

	Mothers									Fathers								
	Total *N* = 228		Intervention *N* = 142			Control *N* = 86			Comparison	Total *N* = 197		Intervention *N* = 121			Control *N* = 76			Comparison
T1 Week 28 of pregnancy	*n*	%	*n*	%	Median *(min‐max)*	*n*	%	Median *(min ‐* *max)*	*p*‐value^*^	*n*	%	*n*	%	Median *(min ‐* *max)*	*n*	%	Median *(min ‐* *max)*	*p*‐value^*^
**Age**	228	100	142	100	31 (22–47)	86	100	30 (22–42)	0.13	195	99.0	120	99.2	32 (22–50)	75	98.7	32 (23–46)	0.87
< 28	46	20.2	26	18.3	−	20	23.3	−	−	30	15.2	15	12.4	−	15	19.7	−	−
29–35	149	65.4	95	66.9	−	54	62.8	−	−	119	60.4	80	66.1	−	39	51.3	−	−
> 36	33	14.5	21	14.8	−	12	14.0	−	−	46	23.4	25	20.7	−	21	27.6	−	−
Missing	0	0	0	0	−	0	0	−	−	2	1.0	1	0.8	−	1	1.3	−	−
**Marital status**	217	95.2	133	93.7	−	84	97.7	−	NA^***^	196	99.5	120	99.2	−	76	100	−	NA^***^
Single/Friend	7	3.1	3	2.1	−	4	4.7	−	−	1	0.5	0	0	−	1	1.3	−	−
Partnered	130	57.0	78	54.9	−	52	60.5	−	−	122	61.9	74	61.2	−	48	63.2	−	−
Married	80	35.1	52	36.6	−	28	32.6	−	−	73	37.1	46	38.0	−	27	35.5	−	−
Missing	11	4.8	9	6.3	−	2	2.3	−	−	1	0.5	1	0.8	−	0	0	−	−
**Education**	227	99.6	141	99.3	−	86	100	−	0.08	196	99.5	120	99.2	−	76	100	−	0.60
Primary/Secondary School	22	9.6	12	8.5	−	10	11.6	−	−	34	17.3	20	16.5	−	14	18.4	−	−
College/University < 4 Years	65	28.5	34	23.9	−	31	36.0	−	−	63	32.0	36	29.8	−	27	35.5	−	−
College/University ≥ 4 Years	140	61.4	95	66.9	−	45	52.3	−	−	99	50.3	64	52.9	−	35	46.1	−	−
Missing	1	0.4	1	0.7	−	0	0	−	−	1	0.5	1	0.8	−	0	0	−	−
**Family income before tax (NOK)**	222	97.4	138	97.2	−	84	97.7	−	**< 0.01**	192	97.5	117	96.7	−	75	98.7	−	**0.04**
≤ 1,000,000	103	45.2	54	38.0	−	49	57.0	−	−	83	42.1	43	35.5	−	40	52.6	−	−
> 1,000,000	119	52.2	84	59.2	−	35	40.7	−	−	109	55.3	74	61.2	−	35	46.1	−	−
Missing	6	2.6	4	2.8	−	2	2.3	−	−	5	2.5	4	3.3	−	1	1.3	−	−
**Nationality**	228	100	142	100	−	86	100	−	1	196	99.5	120	99.2	−	76	100	−	0.58
Norway	196	86.0	122	85.9	−	74	86.0	−	−	152	77.2	91	75.2	−	61	80.3	−	−
Other	32	14.0	20	14.1	−	12	14.0	−	−	44	22.3	29	24.0	−	15	19.7	−	−
Missing	0	0	0	0	−	0	0	−	−	1	0.5	1	0.8	−	0	0	−	−
**Mental health condition** **Previous**	227	99.6	141	99.3	−	86	100	−	.73	196	99.5	120	99.2	−	76	100	−	0.09
Yes	30	13.2	20	14.1	−	10	11.6	−	−	10	5.1	9	7.5	−	1	1.3	−	−
No	197	86.4	121	85.2	−	76	88.4	−	−	186	94.4	111	91.7	−	75	98.7	−	−
Missing	1	0.4	1	0.7	−	0	0	−	−	1	0.5	1	0.8	−	0	0	−	−
**Mental health condition** **Present**	227	99.6	141	99.3	−	86	100	−	1	196	99.5	120	99.2	−	76	100	−	0.68
Yes	9	3.9	6	4.2	−	3	3.5	−	−	6	3.0	3	2.5	−	3	3.9	−	−
No	218	95.6	135	95.1	−	83	96.5	−	−	190	96.5	117	96.7	−	73	96.1	−	−
Missing	1	0.4	1	0.7	−	0	0	−	−	1	0.5	1	0.8	−	0	0	−	−
**Depressive symptoms**	224	98.2	139	97.9	−	85	98.8	−	0.23	193	98.0	119	98.3	−	74	97.4	−	0.68
Yes (EPDS ≥ 10)	40	17.5	21	14.8	−	19	22.1	−	−	6	3.1	3	2.5	−	3	3.9	−	−
No (EPDS < 10)	184	80.7	118	83.1	−	66	76.7	−	−	187	94.9	116	95.9	−	71	93.4	−	−
Missing	4	1.8	3	2.1	−	1	1.2	−	−	4	2.0	2	1.7	−	2	2.6	−	−
**Perception of sleep**	224	98.2	138	97.2	−	86	100	−	**0.03**	193	98.0	117	96.7	−	76	100	−	**0.052** ^**^ (Pearson/Fisher's 0.035/ 0.039)
Enough	138	60.5	77	54.2	−	61	70.9	−	−	147	74.6	83	68.6	−	64	84.2	−	−
Not enough	86	37.7	61	43.0	−	25	29.1	−	−	46	23.4	34	28.1	−	12	15.8	−	−
Missing	4	1.8	4	2.8	−	0	0	−	−	4	2.0	4	3.3	−	0	0	−	−
**Relationship satisfaction**	223	97.8	138	97.2	33 (18–35)	85	98.8	34 (10–35)	0.75	193	98.0	119	98.3	34 (20–35)	74	97.4	34 (23–35)	0.40
Missing	5	2.2	4	2.8	−	1	1.2	−	−	4	2.0	2	1.7		2	2.6	−	−
**Complication during pregnancy**	226	99.1	142	100	−	84	97.7	−	0.36	−	−	−	−	−	−	−	−	−
Yes	53	23.2	30	21.1	−	23	26.7	−	−	−	−	−	−	−	−	−	−	−
No	173	75.9	112	78.9	−	61	70.9	−	−	−	−	−	−	−	−	−	−	−
Missing	2	0.9	0	0	−	2	2.3	−	−	−	−	−	−	−	−	−	−	−
**First‐time father**	−	−	−	−	−	−	−	−	−	196	99.5	120	99.2	−	76	100	−	0.35
Yes	−	−	−	−	−	−	−	−	−	181	91.9	113	93.4	−	68	89.5	−	−
No	−	−	−	−	−	−	−	−	−	15	7.6	7	5.8	−	8	10.5	−	−
Missing	−	−	−	−	−	−	−	−	−	1	0.5	1	0.8	−	0	0	−	−

* Mann‐Whitney *U* test for continuous variables and chi‐square (*χ* ^2^) test for Independence for categorical variables. Reporting continuity correction values for 2 x 2 tables and Fisher's exact test 2‐sided *p*‐values when small numbers (less than 5).

** Since both Pearson's chi‐square (*χ* ^2^) *p*‐value and Fisher's exact test 2‐sided *p*‐value were statistically significant, we included “*Perception of sleep”* regarding fathers in further analyses despite continuity corrected *p*‐value of 0.052.

*** NA = not applicable. Additionally, due to rounding up, sum totals do not always sum up to 100%, but 100.1 or 99.9%.

### 3.2 The Impact of NF Home Visiting Program on PSE

In general, the fathers had a lower level of PSE than the mothers at both time points. However, the level of PSE increased from T2 to T3 for all groups and was overall high, considering the maximum of 80 points, see Table 2.

**TABLE 2:** Level of PSE at T2 and T3 (min. 20 points to max. 80 points).

	Total					Intervention					Control				
Participants at T2 and T3	*n* ^a^	%^b^	*Median* *(min.‐max.)*	*Mean*	*SD*	*n* ^a^	%^b^	*Median* *(min.‐max.)*	*Mean*	*SD*	*n* ^a^	%^b^	*Median* *(min.‐max.)*	*Mean*	*SD*
**Mothers** **T2** *n* = 184	165	89.7	70 (34–80)	69	7.9	107	89.9	70 (34–80)	68.7	8.6	58	89.2	70 (55–80)	69.5	6.5
**Mothers** **T3** *n* = 167	162	97.0	74 (55–80)	72.1	6.8	104	97.2	73 (55–80)	71.4	7.04	58	96.7	75 (57–80)	73.2	6.3
**Fathers** **T2** *n* = 153	139	90.8	66 (43–80)	65.6	7.6	88	89.8	67 (43–80)	66.4	7.5	51	92.7	65 (48–80)	64.2	7.7
**Fathers** **T3** *n* = 135	122	90.4	69 (47–80)	68.1	7.8	74	88.1	69 (47–80)	68.3	7.6	48	94.1	69.5 (50–80)	67.9	8.2

^a^ Participants answering the whole PMP S‐E tool and are included in the analyses.

^b^ % of the participants at T2 or T3 who answered the whole PMP S‐E tool and are included in the analyses.

When adjusted for baseline differences between the groups, fathers in the intervention group had 3.58 (95% CI [0.73 to 6.29]) points higher level of PSE at T2 compared with fathers in the control group. The effect size was small (ES = 0.22), showing a statistically significant difference (*p* < 0.01) between the intervention and the control group; for details, see Table 3.

**TABLE 3:** The impact of NF home visiting program on fathers’ PSE at 6 weeks (T2). The estimates are derived using 1000 bootstrap samples.

Variables	Fathers’ PSE at T2			
	B	95% Cl	*p*‐value	Effect size
Intervention	3.58	0.73 to 6.29	**<0.01**	0.22
Enough sleep (T1)	3.65	0.28 to 6.83	**0.02**	0.19
Income over 1 mill. NOK (T1)	−2.86	−5.60 to −0.35	**0.03**	−0.18

At T3, we found no statistically significant difference in the fathers’ level of PSE between the intervention and control groups (*B* = 1.54; 95% CI [−1.50 to 4.50]; *p* = 0.32), indicating no statistically significant impact of NF compared to the control group.

For mothers, we found no statistically significant difference in the level of PSE when comparing the intervention group with the control group at T2 (*B* = −0.44; 95% CI [−2.73 to 1.93]; *p* = 0.73) or at T3 (*B* = −1.32; 95% CI [−3.43 to 0.97]; *p* = 0.23). Perception of enough sleep at T1 had a statistically significant impact on the level of PSE at T2 (*B* = 3.07; 95% CI = [0.50 to 5.74]; *p* = 0.02). However, the effect size was negligible (ES = 0.18); see Table 4.

**TABLE 4:** The impact of NF home visiting program on mothers’ PSE at 6 weeks (T2). The estimates are derived using 1000 bootstrap samples.

Variables	Mothers’ PSE at T2			
	B	95% Cl	*p*‐value	Effect size
Intervention	−0.44	−2.73 to 1.93	0.73	−0.03
Enough sleep (T1)	3.07	0.50 to 5.74	**0.02**	0.18
Income over 1 mill. NOK (T1)	0.56	−1.86 to 3.04	0.67	0.04

### 3.3 Associations Between Mothers’ PSE at Six Weeks Postnatally (T2) and Depressive Symptoms and Perception of Sleep

The prevalence of depressive symptoms (EPDS ≥ 10) in the whole sample of mothers at T2 was 15.2% (*n* = 28), and about one‐half (*n* = 94, 51.1%) reported a perception of enough sleep. Our association analysis revealed a statistically significant association between PSE and both “depressive symptoms” and “perception of sleep” at T2, while “perception of sleep” assessed at T1 did not reach the level of statistically significance. When bootstrapping the final model, mothers with depressive symptoms showed, on average, 7.59 points (CI [−12.25 to −3.05]; *p* = < 0.01) lower level of PSE compared to mothers who did not report having depressive symptoms. Moreover, mothers who reported enough sleep had 3.19 (CI [0.91 to 5.37]; *p* = < 0.01) points higher level of PSE compared to those who reported not having enough sleep, see Table 5.

**TABLE 5:** Association model between mothers’ PSE (T2) and depressive symptoms (EPDS ≥ 10) (T2) and perception of sleep (T1 and T2).

Model	Independent variables	Mothers’ PSE at T2			
		B	95 % CI	*p*‐value	Effect size
**Backward regression**	Enough sleep (T1)	1.87	−0.43 to 4.17	0.11	0.12
	EPDS ≥ 10 (T2)	−6.88	−10.11 to −3.64	**<0.01**	−0.31
	Enough sleep (T2)	2.91	0.58 to 5.24	**0.02**	0.19
**Final model** with bootstrapped CI (1000 samples)	EPDS ≥ 10 (T2)	−7.59	−12.25 to −3.05	**<0.01**	−0.25
	Enough sleep (T2)	3.19	0.91 to 5.37	**<0.01**	0.21

The sensitivity analyses regarding missing values of the PMP S‐E tool and the possible impact of the Covid‐19 pandemic confirmed the results of the main analyses.

## 4 Discussion

Our main finding was that NF had a statistically significant positive impact on fathers’ PSE at six weeks postnatally, while no impact was found at three months compared to the sample receiving standard care only. In the sample of mothers, no statistically significant differences in PSE were found between the intervention and the control group. Further, we found that both depressive symptoms (EPDS ≥ 10) and perception of enough sleep were associated with mothers’ PSE at six weeks postnatally.

The transition to parenthood is naturally different in fathers and mothers, and fathers are found to have lower levels of PSE than mothers during the first weeks after birth (Fong et al. 2018; Salonen et al. 2009), which is consistent with the findings in our study. The positive impact of NF on fathers’ level of PSE at six weeks postnatally indicates that a home visit during pregnancy and the possibility of additional home visits, if needed, might help to strengthen fathers in their transition to fatherhood. Being included as the non‐birthing partner even before the birth and exposed to topics like *expectations of becoming a parent* and *reflections on one's own childhood* might have supported their preparation for fatherhood, enhanced their connection with the mother, and helped to establish functional coparenting at an earlier stage. This assumption aligns with Solberg et al.’s (2022) findings in their qualitative study of fathers receiving NF. The authors found that the home visit during pregnancy facilitated the inclusion and involvement of fathers in the CHS and provided couples with a shared foundation for further reflection prior to embarking on parenthood. These findings are supported by studies in which both functional coparenting and interventions facilitating coparenting are found to enhance fathers’ PSE (Favez et al. 2016; Feinberg et al. 2020).

Another explanation of the positive impact on fathers’ PSE might be related to predictability of what to expect from the CHS postnatally (Barimani and Vikström 2015), established by receiving information about the service during pregnancy and meeting the PHN who will provide follow‐up care postnatally. This assumption is supported in a qualitative study by Sæther et al. (2024), who found the NF prenatal home visit to facilitate management, as well as relational and informational continuity in a sample of mothers and fathers from the intervention group. In general, the fathers lacked more basic information about the service than the mothers, exemplified by fathers not being aware that a postnatal home visit had been part of standard care through many years. Other recent studies report that fathers lack knowledge on how to use the regular CHS, perceive the service as the women's world, and feel excluded (Høgmo et al. 2021; Solberg and Glavin 2018; Wells 2016). During pregnancy, mothers are followed up by a midwife at the CHS and/or their general practitioner (GP) and are thus already enrolled in a health service. These factors could explain the difference in impact in fathers and mothers and that the fathers benefitted more by receiving the prenatal NF home visit. However, Sæther et al. (2024) highlight the need for more proactive information provision about standard care and NF throughout the first year postnatally to both parents. In general, studies show a need to make information about the CHS provision more available and easier to use and that PHNs are needed to actively encourage the use of their service (Aston et al. 2015; Cowley et al. 2015; McLeish et al. 2021).

We did not find an impact of NF on fathers’ PSE at three months. One explanation could be related to fathers’ return to work and thereby limited exposure to both NF and/or standard care. Most fathers utilize parental leave in the first weeks after birth and at the end of the child's first year. Additionally, fathers generally participate in fewer visits at the clinics than mothers (Kaiser et al. 2022). This applies to fathers in both the intervention and control groups and could make it more difficult to measure differences between the groups at three months.

In the sample of mothers, we did not find an impact of NF at 6 weeks or at 3 months. These null findings might be related to the high level of mothers’ PSE in both groups (Table 2). Leahy‐Warren et al. (2012) report a median of 60 and a mean score of 65.9 in their study of 410 first‐time mothers assessed at six weeks postnatally, which they classified as a high level of PSE. Measuring differences between levels close to the maximum score can be difficult and confounded by a ceiling effect (Polit and Beck 2021).

The development of PSE and the transition to parenthood are complex, with many potential sources of impact (Bandura 1997; Sæther et al. 2023, 2024). The overall high level of PSE in mothers could be because they received sufficient standard care during pregnancy (from midwives and/or GPs) and the first three months postnatally (from CHS, Figure 1). Verbal persuasion from their partner could also be a potential source of the high levels of PSE (Feinberg 2002, 2003; Leahy‐Warren et al. 2012; Sæther et al. 2023, 2024). In this study, both mothers and fathers scored high in relationship satisfaction before entering parenthood. High relationship satisfaction is related to functional coparenting, indicating that parents are more likely to support each other rather than undermine each other (Bandura 1997; Feinberg 2002, 2003).

We find the associations between mothers’ PSE and their reported depressive symptoms and their perception of sleep (Table 5) of clinical interest although the ES are small. Depressive symptoms are described to affect the mother–child relationship negatively and to be an underdiagnosed obstetric complication (Albanese et al. 2019). Low PSE is found to predict a higher risk of developing postnatal depression, and a bidirectional association wherein PSE both affects and is affected by mental health outcomes is identified (Albanese et al. 2019). Leahy‐Warren et al. (2012) describe an inverse relationship between PSE and depressive symptoms, indicating that the higher the level of PSE, the lower the prevalence of depressive symptoms. The negative impact of sleep deprivation on parenting functioning has been reported (McQuillan et al. 2019), and PSE has been found to mediate the relationship between parental fatigue and parental warmth/hostility (Albanese et al. 2019). Based on these findings, addressing depressive symptoms and sleep challenges as topics at the first NF home visit during pregnancy could stimulate the parents to develop a shared understanding of these possible challenges, which in turn can enable them to support each other in the postnatal period. It is important to note that sleep challenges (Da Costa et al. 2021; Sæther et al. 2023, 2024) and depressive symptoms (Cameron et al. 2016; Høgmo et al. 2021) are not solely limited to mothers but might also affect fathers.

### 4.1 Strengths and Limitations

This study generated knowledge regarding the impact of a universal, health‐promoting, and individual‐based home visiting program on PSE in new mothers and fathers. However, there are methodological limitations to be addressed.

The lack of information about those who declined to participate at T1 and the fact that the study was not randomized could have led to a selection bias. Further, the sampled individuals could be characterized as a low‐risk sample concerning socioeconomic variables. Thus, the generalizability of the results might be limited. The primary outcome, PSE, was not assessed at baseline (T1) before the intervention was introduced, constituting another limitation in our measurement of NF's impact. However, comparing PSE before and after birth might also be disputed, as the baby's introduction affects what is measured. Lastly, the PMP S‐E tool has not been validated in a Norwegian setting or for fathers. Nevertheless, Cronbach's alpha values were satisfactory in mothers and fathers at both measure points, indicating good internal consistency.

### 4.2 Recommendation for Further Research

We recommend conducting studies over a longer period than three months and conducting an extensive process evaluation considering fidelity to provide more knowledge with which to evaluate and further develop NF (Skivington et al. 2021). Lastly, we recommend that future research specifically delve into NF's potential to support women with depressive symptoms and sleep challenges and to look deeper into fathers’ needs for support.

### 4.3 Implications for Policy and Practice

The impact of NF on fathers’ PSE at six weeks is significant. Further, we argue that the absence of significant findings in mothers at six weeks and in both parents at three months does not necessarily diminish the importance of providing universal access to NF. Universal access has the potential to involve fathers, support both parents and facilitate functional coparenting, and reach individuals with additional needs, such as postnatal depression symptoms and sleep challenges. This argument is supported by the qualitative studies of NF (Solberg et al. 2022; Sæther et al. 2024) and by the study of Liyana Amin et al. (2018), which highlights universal programs’ opportunities to target fathers and to reach out to parents who are struggling.

## 5 Conclusion

In a socioeconomic low‐risk population, we found a statistically significant positive impact of NF on fathers’ PSE assessed at six weeks postnatally compared to fathers receiving standard care. We consider this finding clinically relevant, as other studies report that fathers are not receiving tailored and sufficient support from standard care. The lack of significant findings of NF on mothers’ PSE may be attributed to mothers’ generally high levels of PSE in both groups. However, as women with depressive symptoms and perception of insufficient sleep had statistically significantly lower PSE at six weeks, we recommend that PHNs should actively address these possible challenges during pregnancy and follow up postnatally within the NF programme.

## Author Contributions

**Design**: Kristin Marie Sæther, Kari Glavin, Milada Cvancarova Hagen, Bettina Holmberg Fagerlund, and Nina Jøranson. **Data collection**: Kristin Marie Sæther, Malene Brekke, Beate Solberg, Kari Glavin, and Anne‐Martha Utne Øygarden. **Statistical analyses**: Kristin Marie Sæther and Milada Cvancarova Hagen (statistician). **The first draft of this manuscript was made by**: Kristin Marie Sæther. **Critical revisions of the manuscript**: All authors.

## Funding

The research project “New Families—Innovation and Development of the Child Health Service in Oslo”, from which the data material for this study is retrieved, was funded by the Research Council of Norway (project code 282167)

## Ethics Statement

The research project “New Families—Innovation and Development of the Child Health Service in Oslo” was approved by the Regional Committees for Medical and Health Research Ethics (reference no: 2018/1378) and the Norwegian Agency for Shared Services in Education and Research (SIKT) (project number: 735207) and is registered in ClinicalTrials.gov https://clinicaltrials.gov/study/NCT04162626. The study was designed and conducted in accordance with the Helsinki Declaration (World Medical Association 2013). Written informed consent to participate in the study was collected from all participants. Participation was voluntary, and participants could withdraw without giving a reason. All data were treated as confidential, and participant anonymity was guaranteed.

## Conflicts of Interest

The authors report there are no competing interests to declare.

## Data Availability

All relevant data are presented in the article. Further records from this study are available from the corresponding author upon reasonable request.
